# Genetic deletion of the 67‐kDa isoform of glutamate decarboxylase alters conditioned fear behavior in rats

**DOI:** 10.1002/2211-5463.13065

**Published:** 2020-12-30

**Authors:** Kazuyuki Fujihara, Takumi Sato, Yoshiki Miyasaka, Tomoji Mashimo, Yuchio Yanagawa

**Affiliations:** ^1^ Departments of Genetic and Behavioral Neuroscience Graduate School of Medicine Gunma University Maebashi Japan; ^2^ Department of Psychiatry and Neuroscience Graduate School of Medicine Gunma University Maebashi Japan; ^3^ Institute of Experimental Animal Sciences Graduate School of Medicine Osaka University Suita Japan; ^4^ Laboratory Animal Research Center Institute of Medical Science the University of Tokyo Japan

**Keywords:** amygdala, CRISPR/Cas9, fear conditioning, GABA, genome editing, inhibitory neurotransmitter

## Abstract

The GABAergic system is thought to play an important role in the control of cognition and emotion, such as fear, and is related to the pathophysiology of psychiatric disorders. For example, the expression of the 67‐kDa isoform of glutamate decarboxylase (GAD67), a GABA‐producing enzyme, is downregulated in the postmortem brains of patients with major depressive disorder and schizophrenia. However, knocking out the *Gad1* gene, which encodes GAD67, is lethal in mice, and thus, the association between *Gad1* and cognitive/emotional functions is unclear. We recently developed *Gad1* knockout rats and found that some of them can grow into adulthood. Here, we performed fear‐conditioning tests in adult *Gad1* knockout rats to assess the impact of the loss of *Gad1* on fear‐related behaviors and the formation of fear memory. In a protocol assessing both cued and contextual memory, *Gad1* knockout rats showed a partial antiphase pattern of freezing during training and significantly excessive freezing during the contextual test compared with wild‐type rats. However, *Gad1* knockout rats did not show any synchronous increase in freezing with auditory tones in the cued test. On the other hand, in a contextual memory specialized protocol, *Gad1* knockout rats exhibited comparable freezing behavior to wild‐type rats, while their fear extinction was markedly impaired. These results suggest that GABA synthesis by GAD67 has differential roles in cued and contextual fear memory.

## Graphical abstract

Inhibitory neurotransmitter GABA is synthesized by two enzymes, the glutamate decarboxylase 67‐kDa isoform (GAD67) and the 65‐kDa isoform (GAD65). Knocking out the *Gad1* gene, which encodes GAD67, is lethal in mice, and therefore, the association between *Gad1* and emotional function was unclear. In the present study, we assessed the impact of *Gad1* knockout on fear expression and fear memory for the first time, using newly generated knockout rats.

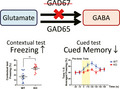

**Abbreviations**
ANOVA: analysis of variance
CRISPR: clustered regularly interspaced short palindrome repeat
CS: conditioned stimulus
GABA: γ‐aminobutyric acid
GAD: glutamate decarboxylase
KO: knockout
US: unconditioned stimulus
WT: wild‐type

Gamma‐aminobutyric acid (GABA) is an inhibitory neurotransmitter in the brain that is thought to be involved in the pathogenesis of neuropsychiatric disorders such as depression [1, 2, 3, 4], anxiety disorders [5, 6], and schizophrenia [7, 8, 9, 10]. GABA is synthesized by enzymes that are independently encoded by two genes, the glutamate decarboxylase 67‐kDa isoform (GAD67) and the 65‐kDa isoform (GAD65) [11, 12, 13]. In rodents, the corresponding genes are *Gad1* and *Gad2*. The expression level of GAD67 is downregulated in the cerebral cortex of patients with major depressive disorder [1] and schizophrenia [14], and this possibly contributes to the neural basis of these disorders. Anxiety and fear, which are common symptoms observed in a wide range of psychiatric disorders, also correlate with the GABAergic system [15, 16]. A decrease in GABA levels in the tissue or blood has been reported in anxiety disorder [5], post‐traumatic stress disorder [17, 18], and mood disorders [19], suggesting that decreased GABA neurotransmission may underlie the neural basis of anxiety in these disorders. This is also consistent with the findings that benzodiazepines, which are stimulants of GABA_A_ receptors, reduce anxiety [20].

The mechanism by which the GABA‐producing system contributes to anxiety and fear has been studied primarily in *Gad2* knockout (KO) mice [15, 21, 22, 23, 24, 25, 26, 27]. This is partly because *Gad1* KO mice exhibit a cleft palate and die on the first postnatal day [28]. In *Gad2* KO mice, phasic inhibition in the basolateral amygdala, which is related to fear memory, is significantly reduced [15]. Consistent with these clinical findings, *Gad2* KO mice exhibit increased anxiety‐like behavior [22] and enhanced active defensive behavior during the fear‐conditioning test [23]. *Gad2* KO mice also display generalized fear [15, 24] and impaired fear extinction [25]. In summary, enhanced fear response or fear memory has been consistently reported in *Gad2* KO mice.

However, the role of *Gad1* (and GAD67) in anxiety and fear is unclear. A study reported that the expression of GAD67 and GAD65 is differentially controlled in fear extinction; GAD67 is upregulated in the prefrontal cortex, while GAD65 is downregulated in the hippocampus during fear memory extinction, suggesting that they have a distinct role in the processing of fear memory [29]. Although fear conditioning is yet to be assessed, *Gad1* ‘heterozygous’ KO mice show anxiety levels comparable to those of wild‐type (WT) mice [30, 31]. It is unclear whether this suggests that GAD67 does not play a significant role in fear and anxiety, or whether a slight reduction in GAD67 levels in heterozygous KO mice is not sufficient to affect their behavior.

We recently obtained a novel tool to address this issue. Using genome editing technology [32], we successfully developed a *Gad1* KO rat line in the Long‐Evans background (Fujihara *et al*. [33]). The perinatal lethality of *Gad1* KO rats was not as severe as that of *Gad1* KO mice; approximately 33% of KO rats could grow up to adulthood. Therefore, in the present study, we characterized the behavioral phenotype of *Gad1* KO rats using the fear‐conditioning test to reveal the role of GAD67 in fear expression, fear memory, and fear extinction.

## Materials and methods

### Animals

*Gad1* KO rats were generated by a previously reported method [32]. The details of the generation procedure are reported elsewhere (Fujihara *et al*. [33]). The animals are available from NBRP‐Rat (https://www.anim.med.kyoto‐u.ac.jp/nbr/Default.aspx). Briefly, the exon 6 of the *Gad1* gene was deleted in the Long‐Evans background using the CRISPR/Cas9 system [34]. The rats used in the present study were obtained by crossing male and female *Gad1* ^+/−^ rats. Only male *Gad1* ^−/−^ (*Gad1* KO) rats were used for behavioral analysis. Littermate *Gad1* ^+/+^ (*Gad1* WT) rats were also used as a control group. Approximately 66% of these rats died at P0, while the remaining 33% survived and were able to grow into adulthood. None of the rats had epileptic seizures during the observation period. Genotyping PCR was performed using the following primers [33]:

5′‐ACTGGGCCATTGTTCCAGCTCCA‐3′,
5′‐GCTCTCTCACGAGTATGCCCTTGCT‐3′,
and 5′‐CGAGCTGGAGAAGGGGGAAGAAGAT‐3′.

The rats were housed in a room maintained at 22 ± 3 °C with a 12‐h light–dark cycle (lights on at 6:00, lights off at 18:00). Food (CLEA Rodent Diet CE‐2, Clea Japan, Meguro, Tokyo, Japan) and water were provided *ad libitum*. All the experiments in this study were approved by the Animal Care and Experimentation Committees of Gunma University and the Animal Research Committee of Osaka University. Every effort was made to minimize the number of animals used and their suffering.

### Western blot analysis

Brain tissue was taken from adult rats, and the S1 fraction was prepared to confirm the *Gad1* knockout. Western blot analysis using a standard protocol was performed as described previously [35]. An antibody that recognizes both GAD65 and GAD67 proteins was used as the primary antibody (1 μg·mL^−1^) [36]. β‐Actin was detected as an internal control using a mouse anti‐β‐actin antibody [1 : 10 000, #AC‐15 (ab6276); Abcam, Cambridge, UK]. The results of the analysis are shown in Fig. S1, and details of the knockout validation are reported elsewhere [33].

### Behavioral analysis

#### Fear‐conditioning test for contextual and cued memory (C‐C experiment)

**Fig. 1:**
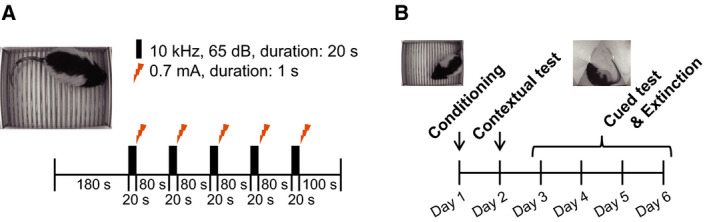
Schematic representation of the fear‐conditioning paradigm for contextual and cued fear memory (C‐C experiment). (A) Following habituation (180 s), 5 tone‐shock pairings were delivered. At the end of the 20‐s tone (conditioned stimulus, CS), an electric shock of 0.7 mA for a duration of 1 s (unconditioned stimulus, US) was delivered. The interval between each tone‐shock pairing was 80 s. After the last shock, rats were left in the conditioning chamber for an additional 100 s. (B) The schedule of the fear‐conditioning test. On the first day of the experiment, we performed a conditioning in (A). On the second day, we performed a contextual test in the same chamber (600 s). From the third day to the sixth day, the same tones as the first day were delivered to the rats in a different chamber with a different shape and context (cued test and fear extinction).

We refer to this experiment as the C‐C (contextual and cued memory) experiment to discriminate it from another experiment. Five‐month‐old rats were used for the analysis (WT, *n* = 12; KO, *n* = 11). We used a modified version of our previous protocol for mice [35]. In the modification process, we also referred to a protocol by Ueno *et al*. [37]. The contextual and cued fear‐conditioning test was conducted in a box surrounded by a sound‐attenuated chamber (O'Hara & Co., Ltd., Tokyo, Japan). This test consisted of a conditioning trial (day 1), a contextual test trial (day 2), and 4‐day cued test trials (day 3 to day 6) (Fig. 1). The rats were placed in a clear acrylic conditioning chamber (33 cm × 25 cm × 28 cm) equipped with a stainless‐steel grid floor connected to an electric shock generator. The brightness of the chamber was 120 lux. On day 1, rats were habituated to the conditioning chamber for 180 s. Immediately after the habituation, they received five auditory tone presentations (CS; 10 kHz, 65 dB, duration: 20 s) with an 80‐s interval, each of which was delivered concomitantly with a foot shock at the end of it (US; 0.7 mA, duration: 1 s). Because the acoustic startle response was comparable between *Gad1* KO and WT rats [33], we can assume that the auditory function is not affected in *Gad1* KO rats. One hundred seconds after the last foot shock, the rats were returned to their home cages (Fig. 1A). A day after the conditioning training, a contextual test (day 2) was performed in the same conditioning chamber for 600 s in the absence of the tone‐shock pairings. Furthermore, a cued test was performed in a novel chamber with different contexts from those of the conditioning chamber to minimize freezing caused by contextual fear conditioning. The test chamber differed from the conditioning chamber in brightness (30 lux), color (black), and shape (triangular prism, 33 cm on one side, and 31.5 cm high). The cued test (day 3) was conducted 24 h after the contextual test. The schedule for the cue test was the same as that for the conditioning, except that no foot shock was administered. In the first 3 min of the exploration period (no CS), we evaluated nonspecific contextual fear. The acquired cued fear was tested through 5 CS periods (no foot shock). On day 4 to day 5, we repeated the same cued test to assess extinction learning (Fig. 1B). The percentage of time that mice exhibited a freezing response (immobility excluding respiration and heartbeat) was measured as an index of fear memory. The data were collected and analyzed using TimeFZ1 (O'Hara & Co., Ltd.). The freezing time was determined automatically by the software every 10 s.

#### Fear‐conditioning test specialized in contextual memory (CTX experiment)

We refer to this experiment as the CTX (contextual memory) experiment to discriminate it from the C‐C experiment. Different cohorts other than the C‐C group were used (WT, *n* = 8; KO, *n* = 8). At least 1 week before the test, we performed an open field test, elevated plus‐maze test, and flinch–jump test as described in the following. We used the same apparatus as the C‐C experiment. This test consisted of a conditioning trial (day 1) and 4‐day contextual test trials (day 2 to day 5) (Fig. 6). In the CTX experiment, we assessed only contextual memory. Therefore, we omitted auditory cues, and the electric shocks were given at random intervals (210, 370, 470, 520, and 615 s from the start of the experiment). The total duration of the experiment on day 1 was the same as that in the C‐C experiment (700 s). The contextual tests (day 2 to day 5) were performed in the same conditioning chamber for 600 s in the absence of electric shocks to evaluate fear extinction.

#### Open field test

A 90 cm × 90 cm open field box (O'Hara) was used for the test. The brightness at the center was 120 lux. Each rat was allowed to walk freely for 10 min in the box. The behavior of the rats was recorded by a CCD camera attached to the ceiling. The distance travelled during a session and the stay time in the central area (25% of the field) were measured using the automated software timeofcr1 (O'Hara).

#### Elevated plus‐maze test

The apparatus consisted of two open arms and two closed arms (O'Hara). The size of each arm was 10 cm × 50 cm. The closed arm had 35‐cm high transparent walls. The arms were placed 50 cm above the floor. The brightness of the center region of the maze was 120 lux. The rats were allowed to walk freely on the entire maze for 10 min. The durations spent in the closed and open arms were measured. The open‐arm ratio was defined as follows: Open‐arm ratio = (the duration spent in the open arm ÷ the total duration spent in the open and closed arms) × 100 (%).

#### Flinch–Jump test

Pain sensitivity can affect the results of the fear‐conditioning test. To determine the sensitivity to the electric shock, we carried out a flinch–jump test based on Lehner *et al*. [38]. Shock naïve rats were given a series of ascending foot shocks from 0.1 to 0.7 mA with a 0.1 mA increment to determine the threshold current for flinch and jump. The interval between shocks was 10 s, and each animal was tested only once. The test was carried out in a different environment from the fear‐conditioning test.

### Statistical analysis

Graphs were generated using graphpad prism (GraphPad Software, Inc., La Jolla, CA, USA). We compared the two genotypes using Welch's *t*‐test and two‐way ANOVA with a simple main effect test. For the ANOVA, we first examined the interaction between the genotype and intraindividual factors. If the interaction was statistically significant, we performed simple main effect tests with Bonferroni correction. If the interaction was not significant, we assessed the main effect of each factor. We also calculated effect size Cohen's *d* for the *t*‐test and partial η‐squared ($\eta_{p}^{2}$) for ANOVA. Welch's *t*‐test and ANOVA were performed using r software (https://www.r‐project.org/) and spss (SPSS Inc., Chicago, IL, USA), respectively. In addition, we calculated a cross‐correlation function between the time course of the tones and freezing to evaluate their synchronization. We also performed analyses using general linear models (GLM) to assess the relationship between two or more parameters. The cross‐correlation function and GLM analyses were calculated using r software. *P*‐values less than 0.05 were considered significant in all statistical tests in the present study.

## Results

### Altered freezing behavior of *Gad1* KO rats during the conditioning process in the C‐C experiment

**Fig. 2:**
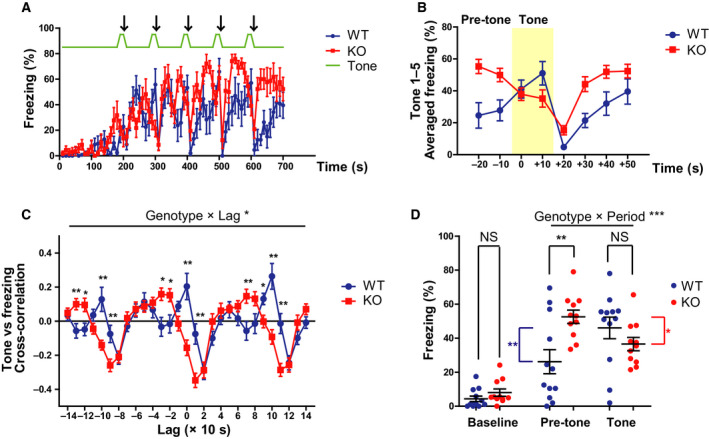
Altered freezing behavior in *Gad1* KO rats during the conditioning process. (A) The time series of freezing behavior during conditioning on day 1 (WT, *n* = 12; KO, *n* = 11). The arrows indicate the timing of the electric shocks. (B) The inverted freezing response of KO rats around the tone‐shock period. The tone period is shaded in yellow. The freezing time courses of all five tone‐shock pairings were averaged to depict the characteristic change in KO rats. (C) Cross‐correlation function between the tone and the freezing time courses. The positive peaks at lag 0 and ±100 s seen in WT rats were undetectable in KO rats. (D) Summary of freezing behavior in each time period. While the freezing behaviors during baseline (0–180 s) and the tone period in KO rats were comparable to those of WT rats, KO rats exhibited significantly excessive freezing during the pretone period. The results are presented as the average ± SEM. Data were analyzed using two‐way ANOVA (C, D) or *t*‐test (D; baseline only). *P < 0.05, **P < 0.01, ***P < 0.001. In (D), red and blue asterisks represent intragroup significant differences. WT, wild‐type; KO, knockout.

We confirmed the loss of GAD67 protein in the whole brain of *Gad1* KO rats using western blot analysis (Fig. S1; Fujihara *et al*. [33]). Then, we performed the fear‐conditioning test, as shown in Fig. 1. On day 1, after 180 s of habituation to the conditioning chamber, the rats were exposed to five tone‐shock pairings (Fig. 1A). During the first 180 s (baseline), no significant difference in freezing time was observed between the two genotypes (Fig. 2A,D; *t* = 1.3622, df = 18.85, *P* = 0.1892, Cohen's *d* = 0.5751). However, the responsiveness to tone‐shock pairings differed between the genotypes (Fig. 2A). To compare the two genotypes more easily, the time courses of freezing around tone‐shock pairings were averaged, as shown in Fig. 2B. WT rats showed increased freezing with the onset of tone, and freezing increased again after a transient decrease caused by the shock (Fig. 2A,B: blue line). In contrast, KO rats showed a partial ‘antiphase’ pattern; higher freezing during the pretone period and decreased freezing with the onset of the tone. Immediately after the shock, the freezing time of KO rats rapidly increased to a higher level than that of WT rats (Fig. 2A,B: red line).

We assessed the above‐mentioned differences using cross‐correlation analysis. Cross‐correlation is a well‐known tool for detecting the synchronicity of neuronal activities [24]. In the present study, we utilized this method to assess the similarity between the tone signal (Fig. 2A: green line) and the time course of the freezing behavior. The cross‐correlation function of WT rats had positive peaks every 100 s around lag = 0 s. This reflected the synchronous increases in the freezing time of WT rats with the tones. However, in KO rats, these positive peaks disappeared and had negative values. This is consistent with a downward trend in freezing with the onset of the tone. The shapes of the cross‐correlation functions of the two genotypes were significantly different (genotype × lag, *F* (28, 588) = 6.628, *P* < 0.001, $\eta_{p}^{2}$ = 0.249), and a simple main effect test revealed several areas that were significantly different between the two groups, mainly at the positive peaks in WT rats (Fig. 2C).

The mean freezing values during the pretone (20 s before the tone) and tone (20 s) periods are summarized in Fig. 2D. There was a significant interaction between genotype and time period (pretone or tone), with a large effect size (genotype × period, *F* (1, 21) = 17.335, *P* < 0.001, $\eta_{p}^{2}$ = 0.452). A simple main effect test showed that pretone freezing was significantly higher in KO rats than in WT rats (*P* = 0.005). In addition, WT rats showed significantly increased freezing from the pretone to tone (*P* = 0.003), while KO rats showed significantly decreased freezing from pretone to tone (*P* = 0.018). The total freezing time during the entire conditioning process was also significantly longer in KO rats than in WT rats, with a large effect size (WT, 25.63 ± 16.27%; KO, 36.74 ± 7.55%; *t* = 2.1278, df = 15.811, *P* = 0.04945, Cohen's *d* = 0.8625). In summary, although responsiveness to the tone was unusual, the fear response itself was increased in KO rats. Furthermore, at the very least, *Gad1* KO rats were considered to have normal hearing as they responded to the tones.

### Increased freezing of *Gad1* KO rats in the contextual test in the C‐C experiment

**Fig. 3:**
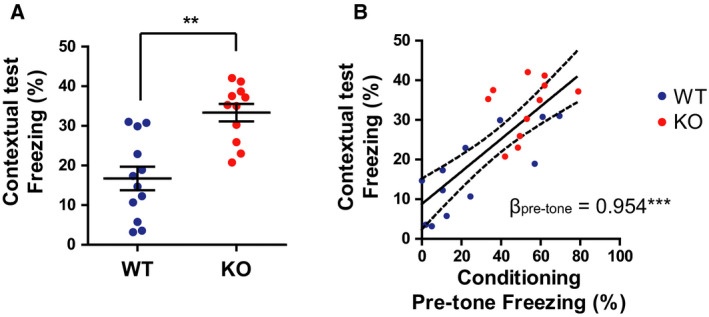
*Gad1* KO rats displayed enhanced freezing in the contextual test (WT, *n* = 12; KO, *n* = 11). (A) Total freezing behavior during the 10‐min contextual test. KO rats displayed significantly higher freezing compared with WT rats. (B) The freezing during the pretone period on day 1 predicted the freezing on the day 2 of contextual test. The solid line and dashed lines represent the regression line and 95% confidence interval of the slope, respectively. β_pretone_ represents the standardized partial regression coefficient. The results are presented as the average ± SEM. Data were analyzed using *t*‐test (A) or general linear model (B).**P < 0.01, ***P < 0.001. WT, wild‐type; KO, knockout.

A contextual test was performed 24 h after the training (Fig. 1B). The total freezing time was significantly prolonged in KO rats compared with that in WT rats, and a very large effect size was observed (Fig. 3A; *t* = 4.498, df = 19.866, *P* < 0.001, Cohen's *d* = 1.8499). The freezing time during the pretone period on day 1 was positively associated with the freezing time during the contextual test (Fig. 3B; standardized regression coefficient *B* = 0.7958, *t* = 6.023, df = 21, *P* < 0.001, adjusted *R^2^* = 0.6159). Considering that the baseline freezing level on day 1 was slightly higher (although not statistically significant) in KO rats with a moderate effect size, the baseline activity might have affected the result on day 2. Thus, we performed multiple regression analyses using the baseline freezing level as a covariate. The standardized partial regression coefficient (β) for the pretone freezing time was still significant (β_pretone_ = 0.9540, *t* = 5.349, df = 20, *P* < 0.001, Adjusted *R^2^* = 0.6279), and that for the baseline freezing was not significant (β_baseline_ = −0.2311, *t* = 1.296, df = 20, *P* = 0.21). These results indicate that pretone freezing during conditioning was a good predictor of freezing in the contextual test; in other words, the fear response on day 1 was recalled in the contextual test.

### Altered fear response of *Gad1* KO rats during the cued test

**Fig. 4:**
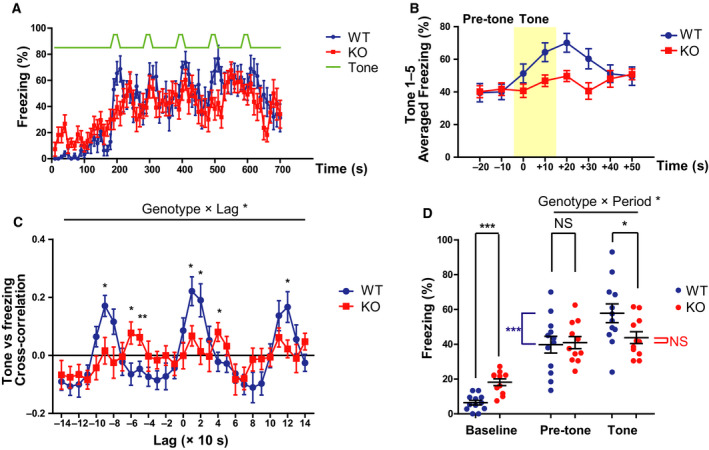
Impaired cued fear memory in *Gad1* KO rats (WT, *n* = 12; KO, *n* = 11). (A) The time series of freezing behavior during the cued test on day 3. (B) The diminished response of *Gad1* KO rats around the tone period. The freezing time courses of all five‐tone periods were averaged to compare WT and KO rats. The tone period is shaded in yellow. (C) Cross‐correlation function between the tone and freezing time courses. The positive peaks at lag 0 and ±100 s observed in WT rats vanished in KO rats. (D) While the baseline freezing (0–180 s) was increased in KO rats, pretone freezing was at the same level as that of WT rats. Remarkably, KO rats did not show an increase in freezing behavior during the tone. The results are presented as the average ± SEM. Data were analyzed using two‐way ANOVA (C, D) or *t*‐test (D; baseline only). *P < 0.05, **P < 0.01, ***P < 0.001; NS, not significant. In (D), blue asterisks or red ‘NS’ represent intragroup statistical comparison. WT, wild‐type; KO, knockout.

We conducted a cued test on day 3 (Fig. 1B). The tone schedule for the cued test was the same as that for the training, except that no foot shock was given. Unlike on day 1, the baseline freezing time was significantly higher in KO rats than in WT rats (Fig. 4A,D; *t* = 5.0168, df = 17.884, *P* < 0.001, Cohen's *d* = 2.1260). Although WT rats showed an increase in freezing time after the tone, the trend was not clear in KO rats (Fig. 4A,B).

Cross‐correlation analysis revealed a peak every 100 s around lag = 0 s in WT rats. This implied an increase in freezing in response to the tone. However, this synchronization was not observed in KO rats. The shape of the cross‐correlation function of the two genotypes was significantly different (genotype × lag, *F* (28, 588) = 2.531, *P* < 0.001, $\eta_{p}^{2}$ = 0.108), and the simple main effect test revealed several areas that were significantly different between the two groups, mainly at the peaks in WT rats (Fig. 4C).

The mean freezing values during the pretone period (20 s before the tone) and during the tone period (20 s) are summarized in Fig. 4D. There was a significant interaction between the genotype and time period (pretone or tone), with a large effect size (genotype × period, *F* (1, 21) = 5.783, *P* = 0.025, $\eta_{p}^{2}$ = 0.216). Freezing during the tone period was significantly lower in KO rats than in WT rats (*P* = 0.042). Although WT rats showed increased freezing when transitioning from pretone to tone (*P* < 0.001), KO rats showed no change (*P* = 0.545).

Although the increase in freezing evoked by auditory cues was lost in *Gad1* KO rats, the average freezing during the tone period significantly increased from the baseline in both genotypes (KO, paired *t*‐test, *t* = 7.0176, df = 10, *P* < 0.001, Cohen's *d* = 2.4253; WT, *t* = 10.885, df = 11, *P* < 0.001, Cohen's *d* = 2.7307). These results suggest that KO rats may also express a certain amount of fear during the tone period.

### Cued fear extinction in *Gad1* KO rats

**Fig. 5:**
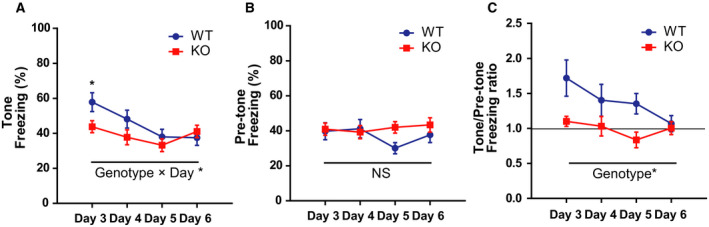
*Gad1* KO rats showed no change in freezing behavior throughout the extinction session compared with WT rats (WT, *n* = 12; KO, *n* = 11). (A) The freezing behavior during the tone of WT rats decreased with repetitive cued tests and became comparable to that of KO rats. (B) The freezing during the pretone periods remained constant in both groups over 4 days. (C) The ratio between the freezing times during tone and during pretone periods also showed the same pattern as (A). Note that the ratio in *Gad1* KO rats was almost 1.0 and unchanged for 4 days. The results are presented as the average ± SEM. Data were analyzed using two‐way ANOVA. *P < 0.05; NS, not significant. WT, wild‐type; KO, knockout.

Repetition of the cued test for a total of 4 days up to day 6 resulted in a decrease in freezing during the tone period in WT rats, but there was no change over time in KO rats (Fig. 5A). We found a significant interaction between the genotype and day (genotype × day, *F* (3, 63) = 2.838, *P* = 0.045, $\eta_{p}^{2}$ = 0.119), and a simple main effect test showed a significant difference between the two groups only on day 3 (*P* = 0.042). However, freezing during pretone was constant in both genotypes (Fig. 5B; genotype, *F* (1, 21) = 0.974, *P* = 0.335, $\eta_{p}^{2}$ = 0.044; day, *F* (3, 63) = 0.874, *P* = 0.460, $\eta_{p}^{2}$ = 0.040; genotype × day, *F* (3, 63) = 1.687, *P* = 0.179, $\eta_{p}^{2}$ = 0.074). When calculating the ratio between the two freezing times (during the tone and pretone periods), the value was still constant and almost 1.0 in KO rats, whereas it decreased from 1.72 ± 0.89 on day 3 to 1.07 ± 0.40 on day 6 in WT rats (Fig. 5C). However, the interaction was not significant in the ANOVA, and only an effect of the genotype and day was found (genotype effect, *F* (1, 21) = 6.702, *P* = 0.017, $\eta_{p}^{2}$ = 0.242; day, *F* (3, 63) = 2.794, *P* = 0.047, $\eta_{p}^{2}$ = 0.117; genotype × day, *F* (3, 63) = 1.493, *P* = 0.225, $\eta_{p}^{2}$ = 0.066).

### Impaired contextual fear extinction of *Gad1* KO rats during the CTX experiment

**Fig. 6:**
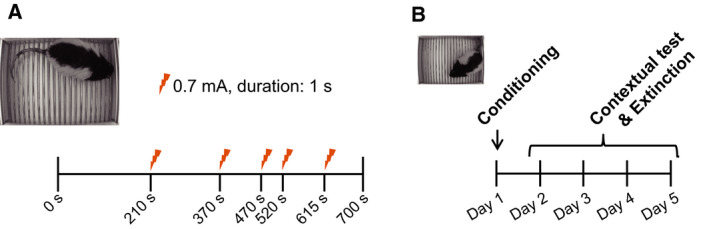
Schematic representation of the fear‐conditioning test specialized for contextual memory (CTX experiment). (A) Following habituation, 5 electric shocks (0.7 mA, 1 s duration) were delivered at random intervals. (B) Schedule of the contextual fear‐conditioning test. On the first day of the experiment, the conditioning shown in (A) was carried out. From the second day to the fifth day, we repeatedly assessed the contextual memory in the same chamber (600 s).

**Fig. 7:**
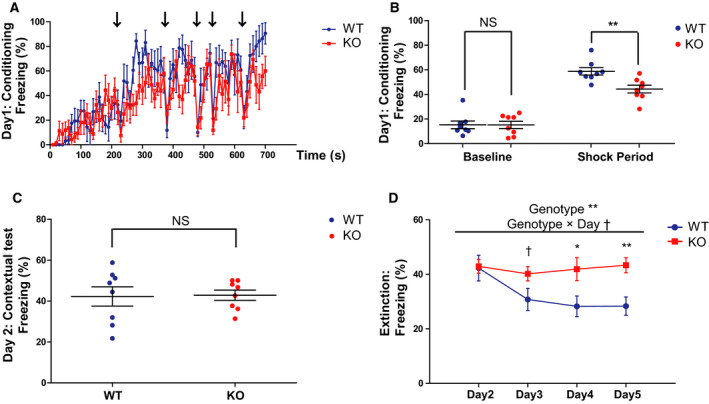
Impaired fear extinction of *Gad1* KO rats in the fear‐conditioning test specialized for contextual memory (CTX experiment). (A) The time series of freezing behavior during conditioning on day 1 (WT, *n* = 8; KO, *n* = 8). The arrows indicate the timing of the electric shocks. (B) Although the baseline freezing (0–180 s) was not significantly different between two genotypes, shock‐induced freezing (210–700 s) in KO rats was lower compared with WT rats on day 1. (C) Total freezing behavior during the 10‐min contextual test on day 2. KO rats and WT rats displayed similar freezing in this protocol. (D) The freezing behavior of WT rats decreased with repetitive contextual tests, while that of KO rats remains unaltered. The results are presented as the average ± SEM. Data were analyzed using *t*‐test (B, C) or two‐way ANOVA (D). ^†^ *P* < 0.1, *P < 0.05, **P < 0.01., ***P < 0.001; NS, not significant. WT, wild‐type; KO, knockout.

Next, we assessed fear extinction in contextual memory. Because the freezing level in the contextual test was relatively low in the C–C experiment (Fig. 3A), we modified the protocol to be specialized for contextual memory in the CTX experiment (Fig. 6). The baseline freezing was similar in both genotypes on day 1 (*t* = 0.0081, df = 13.937, *P* = 0.9937, Cohen's *d* = 0.0040). Although the average freezing time during the shock period (210–700 s) in KO rats was significantly lower than that in WT rats (*t* = 3.3198, df = 13.947, *P* < 0.01, Cohen's *d* = 1.6599), the freezing time in both genotypes increased after the shocks compared with their baseline levels (Fig. 7A,B).

A contextual test was performed 24 h after conditioning, as in the C‐C experiment (Fig. 6A). Unlike in the C‐C experiment, the total freezing time in KO rats was comparable to that in WT rats (Fig. 7C; *t* = 0.1174, df = 10.683, *P* = 0.9087, Cohen's *d* = 0.0587). Repetition of the contextual tests for a total of 4 days up to day 6 resulted in a significant decrease in freezing time in WT rats. On the other hand, KO rats showed no decrease in freezing time for 4 days (Fig. 7D). We found that the genotype and day had significant main effects, and there is a subthreshold interaction between genotype and day (genotype effect, *F* (1, 14) = 6.622, *P* = 0.022, $\eta_{p}^{2}$ = 0.321; day, *F* (3, 42) = 3.341, *P* = 0.028, $\eta_{p}^{2}$ = 0.193; genotype × day, *F* (3, 42) = 2.739, *P* = 0.055, $\eta_{p}^{2}$ = 0.164). Simple main effect tests showed a subthreshold difference on day 3 (*P* = 0.071) and significant differences between the two groups on day 4 (*P* = 0.031) and day 5 (*P* = 0.004). These results indicated that fear extinction of contextual memory was impaired in *Gad1* KO rats.

### Open field test and elevated plus‐maze test

Because locomotor activity and anxiety level can affect fear‐related behaviors, we performed an open field test and elevated plus‐maze test in the same subjects as in the CTX experiment. Although *Gad1* KO rats showed significantly reduced locomotor activity in the open field test, their anxiety‐like behaviors in the elevated plus‐maze test were not altered (Fig. S2). To adjust for possible confounding by the difference in locomotor activity between the two genotypes, we reanalyzed the data of contextual fear extinction using GLMs, which include the distance travelled in the open field test as a covariate. The results are shown in Table S1. Particularly on day 5 in the CTX experiment, the genotype still had a significant effect (*P* < 0.05) after adjusting for the distance travelled. On the other hand, the distance travelled itself had no significant effect on freezing (*P* = 0.8132). Although we should acknowledge the confounding effect of locomotor activity on freezing as a limitation of the present study, these GLM results suggest that the higher level of freezing might be independent of hypoactivity in KO rats.

### Flinch–jump test

Shock sensitivity is also a possible confounding factor in the fear‐conditioning test. We assessed shock sensitivity in the same animals as in the CTX test using the flinch–jump test. The thresholds for flinch and jump were not significantly different between the two genotypes (Table 1).

**Table 1:** Shock sensitivity in *Gad1* KO rats. The threshold currents for flinch and jump are expressed as mean ± SEM (mA). The four right columns are the results of Welch's *t*‐test for group comparison. No significant differences were observed. WT; wild‐type, KO, knockout.

	WT (n = 8)	KO (*n* = 8)	Df	*t*	*P*	*d*
Flinch (mA)	0.238 ± 0.035	0.213 ± 0.028	13.27	0.524	0.61	0.26
Jump (mA)	0.413 ± 0.037	0.375 ± 0.029	13.27	0.74	0.47	0.37

## Discussion

In the present study, we assessed fear expression and fear memory for the first time in *Gad1* KO animals and found the following results. In *Gad1* KO rats, (a) the overall freezing time was prolonged, but (b) the freezing level decreased with the onset of tone during the training in the C‐C experiment. (c) The freezing level was higher than that in WT rats during the contextual test of the C‐C experiment. (d) The baseline freezing was increased in KO rats in the cued test. (e) KO rats displayed no increase in freezing level in response to the tones in the cued test, as if the cued memory was specifically disturbed. (f) KO rats displayed no change in freezing behavior during the fear extinction session in cued fear memory. (g) In the CTX experiment, KO rats displayed slightly lower freezing during the training, while (h) they showed comparable freezing in the contextual test. (i) KO rats showed an impaired contextual fear extinction.

In the C‐C experiment, the responses of *Gad1* KO rats during training for fear‐conditioning and contextual test differed from those of *Gad2* KO mice [15, 21, 22, 23, 24, 25, 26, 27]. Two studies have reported that *Gad2* KO mice exhibited a decreased freezing and increased active defensive behavior and risk assessment behavior during both training and retrieval, as mentioned above [22, 24]. Another study reported a comparable level of freezing between *Gad2* KO mice and WT controls using a different protocol [25]. Unlike these findings, *Gad1* KO rats exhibited a unique pattern of increased freezing during the pretone period and decreased freezing from the pretone level with the onset of the tone. On the other hand, it is of note that *Gad1* KO rats showed lower levels of freezing than WT rats during the training of the CTX experiment as in *Gad2* KO mice. In *Gad2* KO mice, the inhibitory input is impaired in the basolateral amygdala [15]. The basolateral amygdala consists mainly of excitatory neurons and a small population of GABAergic neurons. Hyperexcitability of the basolateral amygdala is associated with pathological anxiety [39]. However, almost all neurons in the central amygdala are GABAergic, and both its inhibition and its excitation cause freezing [39]. These circuit‐level findings are consistent with the higher level of freezing observed in *Gad1* KO rats in the C‐C training, although the reason for these differences in freezing behaviors between *Gad1* KO rats and *Gad2* KO mice is unknown at present. Species differences between mice and rats should be considered, as this can affect the results. *Gad2* knockout rats in the Long‐Evans background are desirable for a direct comparison. GAD65 is known as an activity‐dependent GAD that produces GABA in response to increased neuronal activity in the GABAergic terminal [11]. However, GAD67 is primarily distributed in the soma and constantly produces GABA. At the very least, the present results imply that this baseline production of GABA is also required for regulating an adequate level of fear expression.

*Gad1* KO rats showed abnormally higher freezing in the contextual test of the C‐C experiment, and normal freezing levels in that of the CTX condition. Generally, the freezing level of the contextual test in the CTX experiment is higher than that in the C‐C experiment [40, 41, 42]. This phenomenon is associated with ‘context salience’, which is controlled by hilar perforant path‐associated (HIPP) cells in the dentate gyrus. Under the suppression of HIPP cells, mice show abnormally high freezing in the C‐C condition without affecting freezing in the CTX condition [40]. This phenotype is similar to that observed in *Gad1* KO rats. Because HIPP cells are GABAergic, a deficit in the neurotransmission of the cells might contribute to the phenotype of *Gad1* KO rats.

During the first 180 s of the cued test in the C‐C experiment, *Gad1* KO rats showed remarkably higher freezing than WT rats. Since the freezing behaviors during the baseline period on day 1 did not greatly differ between the two genotypes, we can speculate that the experimental procedure affected the increased freezing on day 3. One possible explanation is fear generalization as seen in *Gad2* KO mice [25], although not necessarily in the same pattern. *Gad2* KO mice display fear generalization to a tone that was not associated with an unconditioned stimulus during training, while *Gad1* KO rats showed higher freezing in the context different from that of training. In any case, these findings suggest that impaired production of GABA is associated with enhanced fear.

Contextual fear memory is dependent on both the hippocampus and the amygdala, while cued fear memory is amygdala‐dependent [43]. The amygdala contains an abundance of GABAergic neurons and forms a complex circuit [39]. This circuit may be the site responsible for the altered response in the cued test observed in *Gad1* KO rats. However, a well‐known lesion study reported that destroying the amygdala caused profound contextual memory deficits simultaneously [43]. Although we should be cautious in drawing conclusions, the peculiar result from the current study may suggest the existence of GABAergic neurons that are largely dependent on GAD67 and are specifically responsible for the association between the tones and electric stimuli. However, cued fear memory was not altered in amygdala‐specific *Gad1* knockdown mice [44], which is inconsistent with our current results. This discrepancy may be attributed to the fact that more than half of the GAD67 proteins remained in the amygdala of the knockdown mice. Due to technical problems, it is still impossible to determine the effects of selective and complete GAD67 elimination in the amygdala. Of course, we should note that there is a possibility that the alteration in freezing in the cued test was due to an alteration in fear expression rather than a deficit in the cued memory itself. Further studies are needed to overcome this limitation of the present experiment.

The contextual fear extinction was severely impaired in *Gad1* KO rats in the present study. Fear extinction is also impaired in amygdala‐specific *Gad1* knockdown mice, although this is assessed in cued memory rather than in contextual memory [44]. Interestingly, parvalbumin neuron‐specific *Gad1* knockdown mice have the same phenotype [45]. Parvalbumin neurons are the largest population in cortical GABAergic neurons [46] and are implicated in the pathogenesis of schizophrenia [9, 35]. In addition, *Gad2* KO mice also show a delay in fear extinction of cued memory [25]. These findings suggest that both GAD67 and GAD65 are crucial for extinction learning.

*Gad1* KO rats are also important in other aspects. Very recently, patients with intellectual disability with a null mutation of *GAD1* were described for the first time [47, 48]. The phenotype of patients differed from *Gad1* KO mice; some patients survived up to 10 years of age, and 30% of patients did not have a cleft palate. These patients were epileptic, whereas no seizures were exhibited in *Gad1* KO rats as far as we have observed. Although the patients also had joint contractures and pes equinovarus, *Gad1* KO rats did not show these comorbidities and are capable of walking, running, and even swimming [33]. Taken together, the severity of the human *GAD1* null mutation can be considered between that of *Gad1* KO mice and *Gad1* KO rats. It is also noteworthy that all known patients with *Gad1* null mutations exhibit mental retardation. It has been postulated that a reduction in GAD67 in the cerebral cortex and hippocampus in schizophrenia may cause cognitive impairment; this is consistent with patients with complete loss of GAD67 function showing mental retardation as a more severe form of cognitive impairment. Considering this, it is also possible that the impaired cued memory found in *Gad1* KO rats reflects a more generalized memory impairment rather than a fear memory‐selective one. Further characterization of the cognitive function of *Gad1* KO rats will be needed.

In summary, the current study provides the first direct evidence that the loss of function of *Gad1* altered conditioned fear behavior in adult rats. Furthermore, it is possible that it could have selectively impaired cued fear memory. Utilizing this novel model animal, further research should be conducted to reveal the differential role of GAD65 and GAD67 in the regulation of fear.

## Conflict of interest

The authors declare no conflict of interest.

## Author contributions

KF and YY designed the research and wrote the manuscript. YM and TM generated *Gad1* KO rats. With help from YM, TM, and YY, KF and TS performed all the other experiments. KF analyzed the data. All authors approved the final manuscript.

## Supporting information


**Fig. S1.** Western blot analysis of GAD67 and GAD65 (whole brain taken from 3‐months‐old rats). GAD67 protein was undetectable in *Gad1* knockout (KO) rat, while GAD65 remains expressed. β‐Actin was also evaluated as an internal control. +/+, wild‐type; +/–, heterozygous *Gad1* KO; –/– homozygous *Gad1* KO.
**Fig. S2.** *Gad1* KO rats showed hypoactivity in open field test and no alterations in anxiety‐like behaviors in elevated plus maze test. a–b Open field test (*n* = 8 for each genotype). (a) *Gad1* KO rats exhibited decreased distance traveled compared with *Gad1* WT rats (*t* (13.735) = 3.0368, *p* < 0.01, Cohen's *d* = 1.518424). (b) The center time of *Gad1* KO rats was not significantly different from that of WT rats (*t* (11.239) = 1.4057, *p* = 0.1869, Cohen's *d* = 0.702844). c–h Elevated plus maze test (*n* = 8 for each genotype). Neither the distance traveled (c), the number of entries into arms (d), the time on open‐arms (e), the time on closed‐arms (f), the time on center (g), nor the open‐arms ratio (h) were significantly different between two genotypes (distance traveled, *t* (10.293) = 0.12119, *p* = 0.9059, Cohen's *d* = 1.518424; number of entries into arms, *t* (13.998) = 1.016, *p* = 0.3269, Cohen's *d* = 0.5080204; time on open‐arms, *t* (13.674) = 0.044551, *p* = 0.9651, Cohen's *d* = 0.02227566; time on closed‐arms, *t* (13.771) = 0.6048, *p* = 0.5551, Cohen's *d* = 0.3024024; time on center, *t* (13.962) = 1.0515, *p* = 0.3109, Cohen's *d* = 0.5257378; open‐arms ratio, *t* (13.778) = 0.34131, *p* = 0.738, Cohen's *d* = 0.1706572). The results are presented as the average ± SEM. * *p* < 0.05, **p < 0.01., ***p < 0.001. WT: wild‐type; KO: knockout; OF: open field; EP: elevated plus maze.
**Table S1.** General linear models describing the relationships between the freezing time on each day and genotype in the CTX experiment. The distance traveled in the open field test was included as a covariate. *β,* standardized partial regression coefficient; *SE*, standard error. †*p* < 0.1, * *p* < 0.05.

## Data Availability

Data will be made available from the corresponding author upon reasonable request.

## References

[1] Karolewicz B , MacIag D , O'Dwyer G , Stockmeier CA , Feyissa AM and Rajkowska G (2010) Reduced level of glutamic acid decarboxylase‐67 kDa in the prefrontal cortex in major depression. Int J Neuropsychopharmacol 13, 411–420. 20236554 10.1017/S1461145709990587 PMC2857696

[2] Gabbay V , Mao X , Klein RG , Ely BA , Babb JS , Panzer AM , Alonso CM and Shungu DC (2012) Anterior cingulate cortex γ‐aminobutyric acid in depressed adolescents: relationship to anhedonia. Arch Gen Psychiatry 69, 139–149. 21969419 10.1001/archgenpsychiatry.2011.131 PMC3711232

[3] Gabbay V , Bradley KA , Mao X , Ostrover R , Kang G and Shungu DC (2017) Anterior cingulate cortex γ‐aminobutyric acid deficits in youth with depression. Transl Psychiatry 7, e1216. 28892070 10.1038/tp.2017.187 PMC5611750

[4] Schür RR , Draisma LWR , Wijnen JP , Boks MP , Koevoets MGJC , Joëls M , Klomp DW , Kahn RS and Vinkers CH (2016) Brain GABA levels across psychiatric disorders: a systematic literature review and meta‐analysis of 1H‐MRS studies. Hum Brain Mapp 37, 3337–3352. 27145016 10.1002/hbm.23244 PMC6867515

[5] Pollack MH , Jensen JE , Simon NM , Kaufman RE and Renshaw PF (2008) High‐field MRS study of GABA, glutamate and glutamine in social anxiety disorder: response to treatment with levetiracetam. Prog Neuropsychopharmacol Biol Psychiatry 32, 739–743. 18206286 10.1016/j.pnpbp.2007.11.023

[6] Long Z , Medlock C , Dzemidzic M , Shin YW , Goddard AW and Dydak U (2013) Decreased GABA levels in anterior cingulate cortex/medial prefrontal cortex in panic disorder. Prog Neuropsychopharmacol Biol Psychiatry 44, 131–135. 23391588 10.1016/j.pnpbp.2013.01.020 PMC3758115

[7] Guidotti A , Auta J , Davis JM , Gerevini VD , Dwivedi Y , Grayson DR , Impagnatiello F , Pandey G , Pesold C , Sharma R *et al* (2000) Decrease in reelin and glutamic acid decarboxylase67 (GAD67) expression in schizophrenia and bipolar disorder: a postmortem brain study. Arch Gen Psychiatry 57, 1061–1069. 11074872 10.1001/archpsyc.57.11.1061

[8] Volk DW , Austin MC , Pierri JN , Sampson AR and Lewis DA (2000) Decreased glutamic acid decarboxylase 67 messenger. Arch Gen Psychiatry 57, 237–245. 10711910 10.1001/archpsyc.57.3.237

[9] Hashimoto T , Volk DW , Eggan SM , Mirnics K , Pierri JN , Sun Z , Sampson AR and Lewis DA (2003) Gene expression deficits in a subclass of GABA neurons in the prefrontal cortex of subjects with schizophrenia. J Neurosci 23, 6315–6326. 12867516 10.1523/JNEUROSCI.23-15-06315.2003 PMC6740534

[10] Curley AA , Arion D , Volk DW , Asafu‐adjei JK , Sampson AR , Fish KN and Lewis DA (2011) Cortical deficits of glutamic acid decarboxylase 67 expression in schizophrenia: clinical, protein, and cell type‐specific features. Am J Psychiatry 168, 921–929. 21632647 10.1176/appi.ajp.2011.11010052 PMC3273780

[11] Obata K (2013) Synaptic inhibition and γ‐aminobutyric acid in the mammalian central nervous system. Proc Japan Acad Ser B 89, 139–156. 23574805 10.2183/pjab.89.139 PMC3669732

[12] Bu DF , Erlander MG , Hitz BC , Tillakaratne NJ , Kaufman DL , Wagner‐McPherson CB , Evans GA and Tobin AJ (1992) Two human glutamate decarboxylases, 65‐kDa GAD and 67‐kDa GAD, are each encoded by a single gene. Proc Natl Acad Sci USA 89, 2115–2119. 1549570 10.1073/pnas.89.6.2115 PMC48607

[13] Soghomonian J and Martin DL (1998) Two isoforms of glutamate decarboxylase: why ? Trends Pharmacol Sci 19, 500–505. 9871412 10.1016/s0165-6147(98)01270-x

[14] Tsubomoto M , Kawabata R , Zhu X , Minabe Y , Chen K , Lewis DA and Hashimoto T (2019) Expression of transcripts selective for GABA neuron subpopulations across the cortical visuospatial working memory network in the healthy state and schizophrenia. Cereb Cortex 29, 3540–3550. 30247542 10.1093/cercor/bhy227 PMC6644854

[15] Lange MD , Jüngling K , Paulukat L , Vieler M , Gaburro S , Sosulina L , Blaesse P , Sreepathi HK , Ferraguti F and Pape HC (2014) Glutamic acid decarboxylase 65: a link between GABAergic synaptic plasticity in the lateral amygdala and conditioned fear generalization. Neuropsychopharmacology 39, 2211–2220. 24663011 10.1038/npp.2014.72 PMC4104340

[16] Müller I , Çalişkan G and Stork O (2015) The GAD65 knock out mouse – a model for GABAergic processes in fear‐ and stress‐induced psychopathology. Genes Brain Behav 14, 37–45. 25470336 10.1111/gbb.12188

[17] Sheth C , Prescot AP , Legarreta M , Renshaw PF , McGlade E and Yurgelun‐Todd D (2019) Reduced gamma‐amino butyric acid (GABA) and glutamine in the anterior cingulate cortex (ACC) of veterans exposed to trauma. J Affect Disord 248, 166–174. 30735853 10.1016/j.jad.2019.01.037

[18] Trousselard M , Lefebvre B , Caillet L , Andruetan Y , de Montleau F , Denis J and Canini F (2016) Is plasma GABA level a biomarker of post‐traumatic stress disorder (PTSD) severity? A preliminary study. Psychiatry Res 241, 273–279. 27208514 10.1016/j.psychres.2016.05.013

[19] Godfrey KEM , Gardner AC , Kwon S , Chea W and Muthukumaraswamy SD (2018) Differences in excitatory and inhibitory neurotransmitter levels between depressed patients and healthy controls: a systematic review and meta‐analysis. J Psychiatr Res 105, 33–44. 30144668 10.1016/j.jpsychires.2018.08.015

[20] Martin JLR , Sainz‐Pardo M , Furukawa TA , Martin‐Sanchez E , Seoane T and Galan C (2007) Review: Benzodiazepines in generalized anxiety disorder: heterogeneity of outcomes based on a systematic review and meta‐analysis of clinical trials. J Psychopharmacol 21, 774–782. 17881433 10.1177/0269881107077355

[21] Kash SF , Johnson RS , Tecott LH , Noebels JL , Mayfield RD , Hanahan D and Baekkeskov S (1997) Epilepsy in mice deficient in the 65‐kDa isoform of glutamic acid decarboxylase. Proc Natl Acad Sci USA 94, 14060–14065. 9391152 10.1073/pnas.94.25.14060 PMC28432

[22] Stork O , Ji FY , Kaneko K , Stork S , Yoshinobu Y , Moriya T , Shibata S and Obata K (2000) Postnatal development of a GABA deficit and disturbance of neural functions in mice lacking GAD65. Brain Res 865, 45–58. 10814732 10.1016/s0006-8993(00)02206-x

[23] Stork O , Yamanaka H , Stork S , Kume N and Obata K (2003) Altered conditioned fear behavior in glutamate decarboxylase 65 null mutant mice. Genes Brain Behav 2, 65–70. 12884963 10.1034/j.1601-183x.2003.00008.x

[24] Bergado‐Acosta JR , Sangha S , Narayanan RT , Obata K , Pape HC and Stork O (2008) Critical role of the 65‐kDa isoform of glutamic acid decarboxylase in consolidation and generalization of Pavlovian fear memory. Learn Mem 15, 163–171. 18323571 10.1101/lm.705408 PMC2275658

[25] Sangha S , Narayanan RT , Bergado‐Acosta JR , Stork O , Seidenbecher T and Pape HC (2009) Deficiency of the 65 kDa isoform of glutamic acid decarboxylase impairs extinction of cued but not contextual fear memory. J Neurosci 29, 15713–15720. 20016086 10.1523/JNEUROSCI.2620-09.2009 PMC6666166

[26] Bergado‐Acosta JR , Müller I , Richter‐Levin G and Stork O (2014) The GABA‐synthetic enzyme GAD65 controls circadian activation of conditioned fear pathways. Behav Brain Res 260, 92–100. 24300892 10.1016/j.bbr.2013.11.042

[27] Müller I , Obata K , Richter‐Levin G and Stork O (2014) GAD65 haplodeficiency conveys resilience in animal models of stress‐induced psychopathology. Front Behav Neurosci 8, 265. 25147515 10.3389/fnbeh.2014.00265 PMC4124590

[28] Asada H , Kawamura Y , Maruyama K , Kume H , Ding R‐G , Kanbara N , Kuzume H , Sanbo M , Yagi T and Obata K (1997) Cleft palate and decreased brain ‐aminobutyric acid in mice lacking the 67‐kDa isoform of glutamic acid decarboxylase. Proc Natl Acad Sci USA 94, 6496–6499. 9177246 10.1073/pnas.94.12.6496 PMC21078

[29] Sangha S , Ilenseer J , Sosulina L , Lesting J and Pape HC (2012) Differential regulation of glutamic acid decarboxylase gene expression after extinction of a recent memory vs. intermediate memory. Learn Mem 19, 194–200. 22511241 10.1101/lm.025874.112

[30] Sandhu KV , Lang D , Müller B , Nullmeier S , Yanagawa Y , Schwegler H and Stork O (2014) Glutamic acid decarboxylase 67 haplodeficiency impairs social behavior in mice. Genes Brain Behav 13, 439–450. 24612522 10.1111/gbb.12131

[31] Nullmeier S , Elmers C , D'Hanis W , Sandhu KVK , Stork O , Yanagawa Y , Panther P and Schwegler H (2020) Glutamic acid decarboxylase 67 haplodeficiency in mice: consequences of postweaning social isolation on behavior and changes in brain neurochemical systems. Brain Struct Funct 225, 1719–1742. 32514634 10.1007/s00429-020-02087-6 PMC7321906

[32] Miyasaka Y , Uno Y , Yoshimi K , Kunihiro Y , Yoshimura T , Tanaka T , Ishikubo H , Hiraoka Y , Takemoto N , Tanaka T *et al* (2018) CLICK: one‐step generation of conditional knockout mice. BMC Genom 19, 318. 10.1186/s12864-018-4713-y PMC5930688 29720086

[33] Fujihara K , Yamada K , Ichitani Y , Kakizaki T , Jiang W , Miyata S , Suto T , Kato D , Saito S , Watanabe M *et al* (2020) CRISPR/Cas9‐engineered Gad1 elimination in rats leads to complex behavioral changes: Implications for schizophrenia. Transl Psychiatry 10, 426. 33293518 10.1038/s41398-020-01108-6 PMC7723991

[34] Cong L , Ran FA , Cox D , Lin S , Barretto R , Habib N , Hsu PD , Wu X , Jiang W , Marraffini LA and Zhang F (2013) Multiplex genome engineering using CRISPR/Cas systems. Science 339, 819–823. 23287718 10.1126/science.1231143 PMC3795411

[35] Fujihara K , Miwa H , Kakizaki T , Kaneko R , Mikuni M , Tanahira C , Tamamaki N and Yanagawa Y (2015) Glutamate decarboxylase 67 deficiency in a subset of GABAergic neurons induces schizophrenia‐related phenotypes. Neuropsychopharmacology 40, 2475–2486. 25904362 10.1038/npp.2015.117 PMC4538341

[36] Hanamura K , Mizui T , Kakizaki T , Roppongi RT , Yamazaki H , Yanagawa Y and Shirao T (2010) Low accumulation of drebrin at glutamatergic postsynaptic sites on GABAergic neurons. Neuroscience 169, 1489–1500. 20600648 10.1016/j.neuroscience.2010.06.043

[37] Ueno M , Yamada K and Ichitani Y (2017) The relationship between fear extinction and resilience to drug‐dependence in rats. Neurosci Res 121, 37–42. 28322983 10.1016/j.neures.2017.03.006

[38] Lehner M , Wisłowska‐Stanek A , Maciejak P , Szyndler J , Sobolewska A , Krzaścik P and Płaźnik A (2010) The relationship between pain sensitivity and conditioned fear response in rats. Acta Neurobiol Exp (Wars) 70, 56–66. 20407487 10.55782/ane-2010-1774

[39] Babaev O , Piletti Chatain C and Krueger‐Burg D (2018) Inhibition in the amygdala anxiety circuitry. Exp Mol Med 50, 18. 29628509 10.1038/s12276-018-0063-8 PMC5938054

[40] Raza SA , Albrecht A , Çallşkan G , Müller B , Demiray YE , Ludewig S , Meis S , Faber N , Hartig R , Schraven B *et al* (2017) HIPP neurons in the dentate gyrus mediate the cholinergic modulation of background context memory salience. Nat Commun 8, 189. 28775269 10.1038/s41467-017-00205-3 PMC5543060

[41] Calandreau L , Jaffard R and Desmedt A (2007) Dissociated roles for the lateral and medial septum in elemental and contextual fear conditioning. Learn Mem 14, 422–429. 17554087 10.1101/lm.531407 PMC1896092

[42] Calandreau L , Desgranges B , Jaffard R and Desmedt A (2010) Switching from contextual to tone fear conditioning and vice versa: the key role of the glutamatergic hippocampal‐lateral septal neurotransmission. Learn Mem 17, 440–443. 20798266 10.1101/lm.1859810

[43] LeDoux JE and Phillips R (1992) Differential contribution of amygdala and hippocampus to cued and contextual fear conditioning. Behav Neurosci 106, 274–285. 1590953 10.1037//0735-7044.106.2.274

[44] Heldt SA , Mou L and Ressler KJ (2012) In vivo knockdown of GAD67 in the amygdala disrupts fear extinction and the anxiolytic‐like effect of diazepam in mice. Transl Psychiatry 2, e181. 23149445 10.1038/tp.2012.101 PMC3565763

[45] Brown JA , Ramikie TS , Schmidt MJ , Báldi R , Garbett K , Everheart MG , Warren LE , Gellért L , Horváth S , Patel S *et al* (2015) Inhibition of parvalbumin‐expressing interneurons results in complex behavioral changes. Mol Psychiatry 20, 1499–1507. 25623945 10.1038/mp.2014.192 PMC4516717

[46] Tamamaki N , Yanagawa Y , Tomioka R , Miyazaki JI , Obata K and Kaneko T (2003) Green fluorescent protein expression and colocalization with calretinin, parvalbumin, and somatostatin in the GAD67‐GFP knock‐in mouse. J Comp Neurol 467, 60–79. 14574680 10.1002/cne.10905

[47] Chatron N , Becker F , Morsy H , Schmidts M , Hardies K , Tuysuz B , Roselli S , Najafi M , Alkaya DU , Ashrafzadeh F *et al* (2020) Bi‐allelic GAD1 variants cause a neonatal onset syndromic developmental and epileptic encephalopathy. Brain 143, 1447–1461. 32282878 10.1093/brain/awaa085 PMC7241960

[48] Neuray C , Maroofian R , Scala M , Sultan T , Pai GS , Mojarrad M , El Khashab H , DeHoll L , Yue W , Alsaif HS *et al* (2020) Early‐infantile onset epilepsy and developmental delay caused by bi‐allelic GAD1 variants. Brain 143, 2388–2397. 32705143 10.1093/brain/awaa178 PMC7447512

