# Supplementary material for: Genetic deletion of the 67‐kDa isoform of glutamate decarboxylase alters conditioned fear behavior in rats
Source: FEBS Open Bio. 2020 Dec 30;11(2):340–53. doi: 10.1002/2211-5463.13065 (PMC7876494; doi:10.1002/2211-5463.13065)
Supplement: Supplementary file 1 — Fig. S1. Western blot analysis of GAD67 and GAD65 (whole brain taken from 3‐months‐old rats). GAD67 protein was undetectable in Gad1 knockout (KO) rat, while GAD65 remains expressed. β‐Actin was also evaluated as an internal control. +/+, wild‐type; +/–, heterozygous Gad1 KO; –/– homozygous Gad1 KO. Fig. S2. Gad1 KO rats showed hypoactivity in open field test and no alterations in anxiety‐like behaviors in elevated plus maze test. a–b Open field test (n = 8 for each genotype). (a) Gad1 KO rats exhibited decreased distance traveled compared with Gad1 WT rats (t (13.735) = 3.0368, p < 0.01, Cohen's d = 1.518424). (b) The center time of Gad1 KO rats was not significantly different from that of WT rats (t (11.239) = 1.4057, p = 0.1869, Cohen's d = 0.702844). c–h Elevated plus maze test (n = 8 for each genotype). Neither the distance traveled (c), the number of entries into arms (d), the time on open‐arms (e), the time on closed‐arms (f), the time on center (g), nor the open‐arms ratio (h) were significantly different between two genotypes (distance traveled, t (10.293) = 0.12119, p = 0.9059, Cohen's d = 1.518424; number of entries into arms, t (13.998) = 1.016, p = 0.3269, Cohen's d = 0.5080204; time on open‐arms, t (13.674) = 0.044551, p = 0.9651, Cohen's d = 0.02227566; time on closed‐arms, t (13.771) = 0.6048, p = 0.5551, Cohen's d = 0.3024024; time on center, t (13.962) = 1.0515, p = 0.3109, Cohen's d = 0.5257378; open‐arms ratio, t (13.778) = 0.34131, p = 0.738, Cohen's d = 0.1706572). The results are presented as the average ± SEM. * p < 0.05, **p < 0.01., ***p < 0.001. WT: wild‐type; KO: knockout; OF: open field; EP: elevated plus maze. Table S1. General linear models describing the relationships between the freezing time on each day and genotype in the CTX experiment. The distance traveled in the open field test was included as a covariate. β, standardized partial regression coefficient; SE, standard error. †p < 0.1, * p < 0.05. [file FEB4-11-340-s001.pdf]

Supporting Information

Genetic Deletion of Glutamate Decarboxylase 67-kDa Isoform Alters Conditioned Fear Behavior in Rats

Kazuyuki Fujihara[1,2,*], Takumi Sato[1], Yoshiki Miyasaka[3], Tomoji Mashimo[4], and Yuchio Yanagawa[1,*]

[1] Departments of Genetic and Behavioral Neuroscience, Graduate School of Medicine, Gunma University, 371-8511 Maebashi, Gunma, Japan

[2] Department of Psychiatry and Neuroscience, Graduate School of Medicine, Gunma University, 371-8511 Maebashi, Gunma, Japan

[3] Institute of Experimental Animal Sciences, Graduate School of Medicine, Osaka University 565-0871 Suita, Osaka, Japan

[4] Laboratory Animal Research Center, Institute of Medical Science, the University of Tokyo 108-8639 Tokyo, Japan

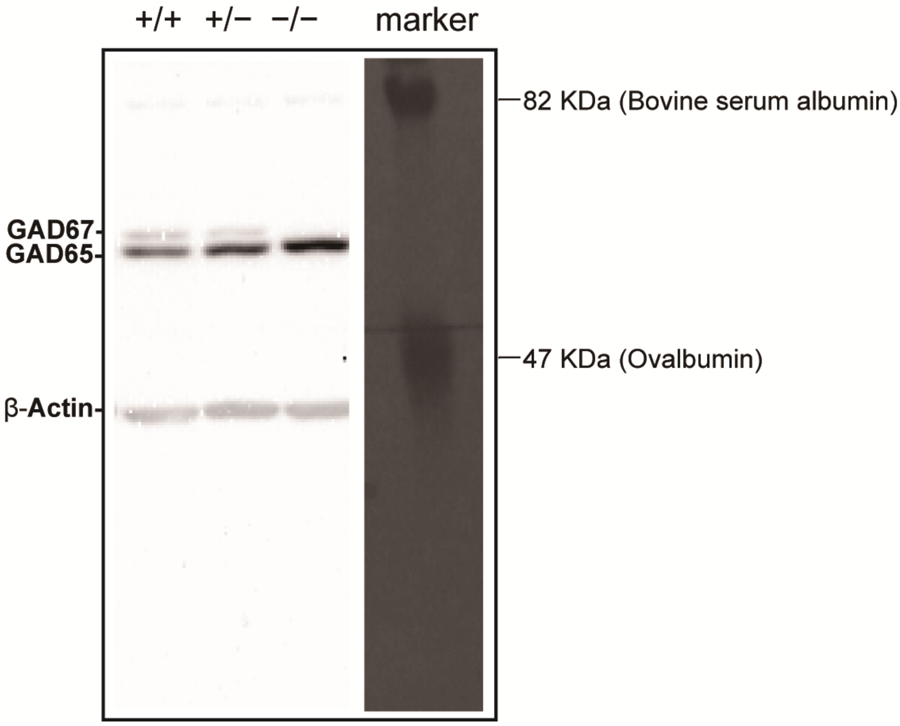

Supplementary Figure S1. Western blot analysis of GAD67 and GAD65 (whole brain taken from 3-months-old rats). GAD67 protein was undetectable in *Gad1* knockout (KO) rat, while GAD65 remains expressed. β-Actin was also evaluated as an internal control. +/+, wild-type; +/–, heterozygous *Gad1* KO; –/– homozygous *Gad1* KO.

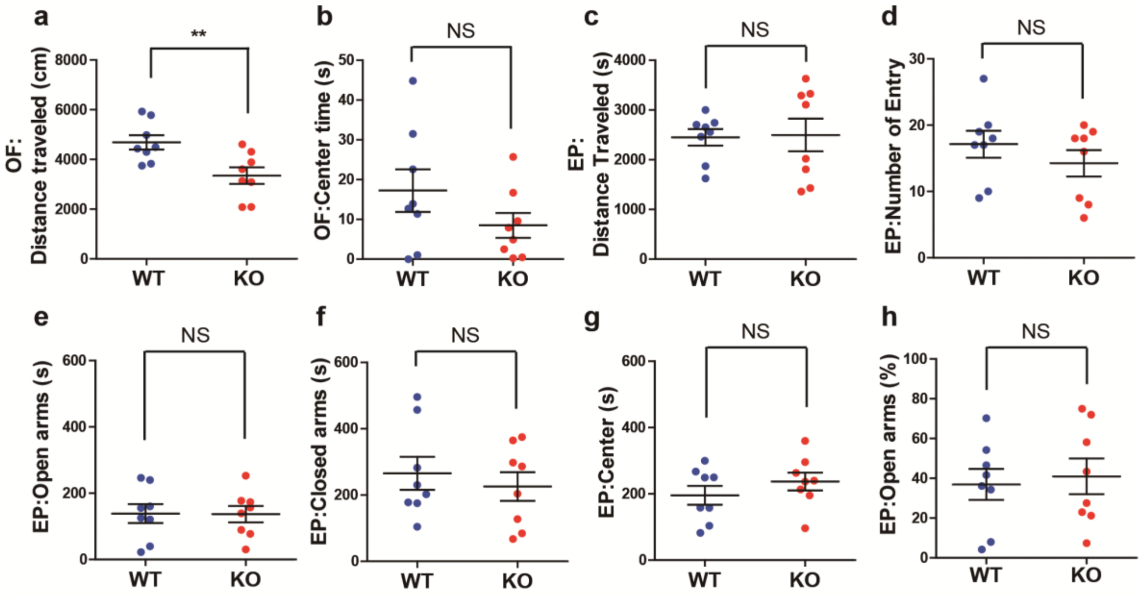

Supplementary Figure S2. *Gad1* KO rats showed hypoactivity in open field test and no alterations in anxiety-like behaviors in elevated plus maze test. a–b Open field test (n = 8 for each genotype). (a) *Gad1* KO rats exhibited decreased distance traveled compared with *Gad1* WT rats (t (13.735) = 3.0368, p < 0.01, Cohen's d = 1.518424). (b) The center time of *Gad1* KO rats was not significantly different from that of WT rats (t (11.239) = 1.4057, p = 0.1869, Cohen's d = 0.702844). c–h Elevated plus maze test (n = 8 for each genotype). Neither the distance traveled (c), the number of entries into arms (d), the time on open-arms (e), the time on closed-arms (f), the time on center (g), nor the open-arms ratio (h) were significantly different between two genotypes (distance traveled, t (10.293) = 0.12119, p = 0.9059, Cohen's d = 1.518424; number of entries into arms, t (13.998) = 1.016, p = 0.3269, Cohen's d = 0.5080204; time on open-arms, t (13.674) = 0.044551, p = 0.9651, Cohen's d = 0.02227566; time on closed-arms, t (13.771) = 0.6048, p = 0.5551, Cohen's d = 0.3024024; time on center, t (13.962) = 1.0515, p = 0.3109, Cohen's d = 0.5257378; open-arms ratio, t (13.778) = 0.34131, p = 0.738, Cohen's d = 0.1706572). The results are presented as the average ± SEM. * p < 0.05, **p < 0.01., ***p < 0.001. WT: wild-type; KO: knockout; OF: open field; EP: elevated plus maze.

a. CTX: day 2

	β	*βSE*	*t*	*p*	
Intercept	0.000	0.276	0	1	
genotype	–0.1064	0.367	–0.29	0.777	
distance traveled	0.114	0.356	0.32	0.754	

R-squared: 0.008784, Adjusted R-squared: –0.1437

F (2, 13) = 0.0576 , p = 0.9443

b. CTX: day 3

	β	*βSE*	*t*	*p*	
Intercept	0.000	0.246	0	1	
genotype	–0.4545	0.327	–1.39	0.188	
distance traveled	–0.0356	0.317	–0.112	0.912	

Multiple R-squared: 0.2147, Adjusted R-squared: 0.09386

F (2, 13) = 1.777 , p = 0.2079

c. CTX: day 4

	β	*βSE*	*t*	*p*	
Intercept	0.000	0.233	0	1	
genotype	–0.5506	0.310	–1.774	0.0994	†
distance traveled	–0.01219	0.300	–0.041	0.9683	

Multiple R-squared: 0.2926, Adjusted R-squared: 0.1837

F (2, 13) = 2.688, p = 0.1054

d. CTX: day 5

	β	*βSE*	*t*	*p*	
Intercept	0.000	0.203	0	1	
genotype	–0.6596	0.270	–2.439	**0.0298**	*
distance traveled	–0.06314	0.262	–0.241	0.8132	

Multiple R-squared: 0.4627, Adjusted R-squared: 0.3801

F (2, 13) = 5.598, p = **0.01763***

Supplementary Table S1. General linear models describing the relationships between the freezing time on each day and genotype in the CTX experiment. The distance traveled in the open field test was included as a covariate. *β,* standardized partial regression coefficient; *SE,* standard error. †p < 0.1, * p < 0.05.
